# High-level expression of aryl-alcohol oxidase 2 from *Pleurotus eryngii* in *Pichia pastoris* for production of fragrances and bioactive precursors

**DOI:** 10.1007/s00253-020-10878-4

**Published:** 2020-09-19

**Authors:** Nina Jankowski, Katja Koschorreck, Vlada B. Urlacher

**Affiliations:** Institute of Biochemistry, Heinrich-Heine-University Düsseldorf, Universitätsstraße 1, 40225 Düsseldorf, Germany

**Keywords:** Aryl-alcohol oxidase, *Pichia pastoris* (*Komagataella phaffii*), Flavoprotein, Aromatic alcohols, Fragrances, Piperonal

## Abstract

**Abstract:**

The fungal secretome comprises various oxidative enzymes participating in the degradation of lignocellulosic biomass as a central step in carbon recycling. Among the secreted enzymes, aryl-alcohol oxidases (AAOs) are of interest for biotechnological applications including production of bio-based precursors for plastics, bioactive compounds, and flavors and fragrances. Aryl-alcohol oxidase 2 (*Pe*AAO2) from the fungus *Pleurotus eryngii* was heterologously expressed and secreted at one of the highest yields reported so far of 315 mg/l using the methylotrophic yeast *Pichia pastoris* (recently reclassified as *Komagataella phaffii*). The glycosylated *Pe*AAO2 exhibited a high stability in a broad pH range between pH 3.0 and 9.0 and high thermal stability up to 55 °C. Substrate screening with 41 compounds revealed that *Pe*AAO2 oxidized typical AAO substrates like *p*-anisyl alcohol, veratryl alcohol, and *trans,trans*-2,4-hexadienol with up to 8-fold higher activity than benzyl alcohol. Several compounds not yet reported as substrates for AAOs were oxidized by *Pe*AAO2 as well. Among them, cumic alcohol and piperonyl alcohol were oxidized to cuminaldehyde and piperonal with high catalytic efficiencies of 84.1 and 600.2 mM^−1^ s^−1^, respectively. While the fragrance and flavor compound piperonal also serves as starting material for agrochemical and pharmaceutical building blocks, various positive health effects have been attributed to cuminaldehyde including anticancer, antidiabetic, and neuroprotective effects. *Pe*AAO2 is thus a promising biocatalyst for biotechnological applications.

**Key points:**

• *Aryl-alcohol oxidase PeAAO2 from P. eryngii was produced in P. pastoris at 315 mg/l.*

• *Purified enzyme exhibited stability over a broad pH and temperature range.*

• *Oxidation products cuminaldehyde and piperonal are of biotechnological interest.*

Graphical abstract
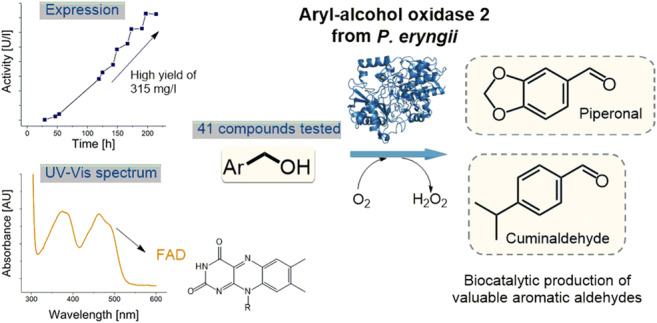

**Electronic supplementary material:**

The online version of this article (10.1007/s00253-020-10878-4) contains supplementary material, which is available to authorized users.

## Introduction

The pursuit of a sustainable and bio-based society includes the search for and development of environmentally friendly production routes for fine chemicals. As a result, more and more biocatalytic processes for production of fine chemicals and valuable building blocks are coming into the focus of research and industry. In green chemistry, the use of biocatalysts has many advantages over conventional organic chemical synthesis, including mild reaction conditions (aqueous systems, ambient temperatures, atmospheric pressure), use of catalyst in non-stoichiometric quantities, and reduced waste production (Sheldon and Woodley 2018). Aryl-alcohol oxidases (AAOs, EC 1.1.3.7) are FAD-dependent oxidoreductases secreted by wood-decaying fungi as glycoproteins (Sannia et al. 1991; Varela et al. 2000a, b). They catalyze the oxidation of primary aromatic and aliphatic polyunsaturated alcohols to the corresponding aldehydes while reducing molecular O_2_ to H_2_O_2_ (Guillén et al. 1992). In some cases, the generated aldehydes can be further oxidized to the aromatic acids depending on the degree of hydration via *gem*-diol formation of the aldehyde (Ferreira et al. 2010). AAOs offer great potential for application in biocatalytic processes, as they only require molecular oxygen for substrate oxidation and generate hydrogen peroxide as byproduct, without the need of added cofactors. In nature, AAOs play an essential role in degradation of lignocellulosic biomass and hence also in carbon recycling. Wood-decaying fungi secrete a whole bunch of oxidative enzymes like laccases, ligninolytic peroxidases, and aryl-alcohol oxidases in order to break down lignin, the most recalcitrant component of lignocellulose (Kirk and Farrell 1987).

While laccases (EC 1.10.3.2) and ligninolytic peroxidases (EC 1.11.1.x) have been intensively studied and applied in different fields including food, textile and cosmetics industry, biorefineries, and bioremediation (Arregui et al. 2019; Falade et al. 2016, 2018; Fillat et al. 2017; Rodríguez Couto and Toca Herrera 2006; Stanzione et al. 2020), H_2_O_2_-producing oxidases like aryl-alcohol oxidases only slowly step forward into biocatalytic applications. For example, an AAO from *Pleurotus eryngii* ATCC 90787 (*Pe*AAO) was studied for production of 2,5-furandicarboxylic acid (FDCA), a bio-based precursor for plastics (Carro et al. 2014; Karich et al. 2018; Serrano et al. 2019a; Viña-Gonzalez et al. 2020). Structure-guided mutagenesis was applied on *Pe*AAO to construct enzyme variants capable of selectively oxidizing secondary aromatic alcohols like (*S*)-1-(*p*-methoxyphenyl)-ethanol to the corresponding ketones (Serrano et al. 2019b; Viña-Gonzalez et al. 2019). This enables the use of AAO in kinetic deracemization of secondary alcohols and generation of enantiomer enriched preparations, which are essential building blocks in the production of pharmaceuticals (Patel 2018). The most recent studies regarding enzyme engineering of AAOs and potential applications were summarized by Viña-Gonzalez and Alcalde (2020).

In general, most oxidation products of AAO-catalyzed reactions have considerable importance for the flavor and fragrance industry. Recently, *Pe*AAO from *P. eryngii* was employed for the conversion of *trans*-2-hexenol to the aldehyde *trans*-2-hexenal, which is of interest for the flavor and fragrance industry as fresh and fruity note of different vegetables and fruits (de Almeida et al. 2019; Van Schie et al. 2018). To gain access to a wider range of pleasant-smelling aldehydes and valuable building blocks via biocatalysis, more information about the substrate scope of AAOs is needed.

One of the factors limiting a broader application and protein engineering of AAOs is their “difficult” expression in recombinant hosts. For instance, the most studied aryl-alcohol oxidase *Pe*AAO from *P. eryngii* yielded only 3 mg/l in *Aspergillus nidulans* (Ferreira et al. 2005). The same enzyme was produced in *Escherichia coli* as inclusion bodies (Ruiz-Dueñas et al. 2006) and yielded 45 mg/l after in vitro refolding*.* However, due to the lack of glycosylation, the *E. coli*-derived recombinant *Pe*AAO showed lower pH and thermal stability than the recombinant enzyme expressed in *A. nidulans* (Ruiz-Dueñas et al. 2006). Efforts were made to optimize *Pe*AAO for secretion in eukaryotic hosts. The optimized *Pe*AAO variant FX7 was constructed using the mutagenic organized recombination process by homologous in vivo grouping (MORPHING) for improved expression in *Saccharomyces cerevisiae* and yielded 2 mg/l of active hyperglycosylated enzyme (Viña-Gonzalez et al. 2015). This variant was further optimized by in vivo shuffling with other *Pe*AAO variants and by the targeted MORPHING of the chimeric signal peptide, which eventually led to the variant FX9. This variant was transferred to *Pichia pastoris* for high-level production, leading to 25.5 mg/l of enzyme (Viña-Gonzalez et al. 2018). Using a basidiomycete as expression host, an AAO from *Pleurotus sapidus* was heterologously produced in *Coprinopsis cinerea* with a yield of 1.4 mg/l (Galperin et al. 2016). In order to fully elucidate fungal AAOs promising properties as biocatalysts in biotechnological processes, a high-yield expression system needs to be established.

Here, we report on high-yield expression of aryl-alcohol oxidase 2 from *P. eryngii* P34 (*Pe*AAO2) in the methylotrophic yeast *P. pastoris* for biotechnological applications. *Pe*AAO2 was characterized and the activity towards a large set of aromatic, heterocyclic, and aliphatic alcohols was investigated. Several compounds not yet described as substrates for AAOs were oxidized by *Pe*AAO2 to furnish important products for the flavor and fragrance industry, and bioactive compounds like piperonal and cuminaldehyde. Furthermore, the influence of glycosylation on enzyme stability was investigated, and kinetic parameters were determined for selected substrates to assess the biotechnological potential of this AAO.

## Materials and methods

### Materials

All chemicals were purchased from abcr GmbH (Karlsruhe, Germany), Acros Organics (Geel, Belgium), Alfa Aesar (Kandel, Germany), AppliChem GmbH (Darmstadt, Germany), BLDpharm (Shanghai, China), Carbolution Chemicals GmbH (St. Ingbert, Germany), Carl Roth GmbH + Co. KG (Karlsruhe, Germany), Fluorochem (Hadfield, UK), J&K Scientific (Lommel, Belgium), Sigma-Aldrich (Schnelldorf, Germany), TCI Chemicals (Eschborn, Germany), and VWR (Darmstadt, Germany). Enzymes and kits were obtained from New England Biolabs (Frankfurt am Main, Germany), Thermo Fisher Scientific (Bremen, Germany), SERVA Electrophoresis GmbH (Heidelberg, Germany), and Zymo Research (Freiburg, Germany).

### Bacterial and yeast strains

*Escherichia coli* strain DH5α used for plasmid amplification was obtained from Clontech Laboratories Inc. (Heidelberg, Germany). *Pichia pastoris* strain X-33 (recently reclassified as *Komagataella phaffii*) used for expression was purchased from Invitrogen (Carlsbad, USA).

### Generation of recombinant *P. pastoris* X-33 transformants

The gene encoding for *Pe*AAO2 from the *P. eryngii* strain P34 (GenBank accession number GU444001.1) was identified by protein BLAST search, using the AAO from the *P. eryngii* strain ATCC 90787 (GenBank accession number AAC72747) as query. The gene *peaao2* was codon optimized (GenBank accession number MT711371) for the expression in *Saccharomyces cerevisiae* using the online tool JCat (Grote et al. 2005). The optimized gene carrying the native signal sequence was synthesized by BioCat GmbH (Heidelberg, Germany) and readily ligated into pPICZA vector (Invitrogen, Carlsbad, USA) employing the restriction sites *Eco*RI and *Not*I, to generate the plasmid pPICZA_*peAAO2*. Chemically competent *E. coli* DH5α cells were transformed with the desired plasmid and transformants were selected on low salt lysogeny broth agar plates (LB; 1% peptone, 0.5% yeast extract, 0.5% NaCl, 1.5% agar) containing 25 μg/ml zeocin (InvivoGen, San Diego, USA). A total of 5 ml of LB medium with 25 μg/ml zeocin was inoculated with transformed *E. coli* cells and cultivated overnight (37 °C and 180 rpm). The plasmids were isolated using the ZR Plasmid Miniprep Kit (Zymo Research, Irvine, USA) according to manufacturer’s instructions.

The isolated plasmid pPICZA_*Pe*AAO2 was linearized in the 5’AOX1 region with FastDigest *Mss*I (Thermo Fisher Scientific, Waltham, USA) and used for transformation of electrocompetent *P. pastoris* X-33 cells. Recombinant *P. pastoris* X-33 cells were selected on yeast extract peptone dextrose sorbitol agar plates (YPDS; 1% yeast extract, 2% peptone, 2% dextrose, 1 M sorbitol, 2% agar) supplemented with 100 μg/ml of zeocin and grown for 4–6 days at 30 °C.

### Enzyme production in shaking flasks

Several *P. pastoris* transformants with pPICZA_*peAAO2* integrated into the genome were used for expression in 100 ml shaking flasks. Precultures were grown overnight (30 °C, 200 rpm) in 10 ml of buffered complex glycerol medium (BMGY; 1% yeast extract, 2% peptone, 100 mM potassium phosphate buffer pH 6.0, 1.34% yeast nitrogen base without amino acids, 4 × 10^−5^% biotin, 1% glycerol). The precultures were used to inoculate 10 ml of buffered complex methanol medium (BMMY; same as BMGY but without glycerol) to an optical density at 600 nm (OD_600_) of 1. The cells were cultivated for 2 days (25 °C, 200 rpm) with the addition of 0.5% (v/v) methanol every 24 h. The OD_600_ and volumetric activity in the cell-free supernatant towards veratryl alcohol were assayed daily as described below.

### Enzyme production in 7.5 l bioreactor

The best producing *P. pastoris* transformant was used for fed-batch cultivation in a 7.5 l bioreactor (Infors, Bottmingen, Switzerland). A total of 3 l of fermentation basal salts medium (per 1 l: 0.47 g CaSO_4_ x 2 H_2_O, 8 ml H_3_PO_4_ (85%)_,_ 9.1 g K_2_SO_4_, 4.2 g KOH, 3.66 g MgSO_4_, 43.5 g glycerol (100%), 0.87 mg biotin, 4.35 ml *Pichia* trace metals (per 1 l of PTM_1_ solution: 6 g CuSO_4_ x 5 H_2_O, 0.08 g NaI, 3 g MnSO_4_. H_2_O, 0.5 g CoCl_2_, 20 g ZnCl_2_, 0.02 g H_3_BO_3_, 0.2 g Na_2_Mo_4_ x 2 H_2_O, 65 g FeSO_4_, 7 H_2_O, 0.2 g biotin, 5 ml H_2_SO_4_)) was inoculated to an OD_600_ of 0.5 from a preculture in 200 ml BMGY medium containing 100 μg/ml zeocin grown over night (30 °C, 200 rpm). For this, the necessary amount of cells was harvested from the preculture by centrifugation (1500x*g*, 5 min, 4 °C) and resuspended in sterile 0.9% sodium chloride solution for inoculation of the fermentation medium. Oxygen was supplied with a rate of 3 l/min and the stirring rate was 800 rpm. The pH was kept at pH 5.0 by titrating 10% phosphoric acid or 25% ammonium hydroxide and the temperature was set to 30 °C. After full consumption of glycerol, a pO_2_-spike controlled fed-batch started with methanol as inducer and sole carbon source. Methanol was added automatically to 0.5% (v/v; with 12 g/l PTM_1_ solution) when a sharp increase in pO_2_ indicated depletion of the carbon source. After induction, the temperature was reduced to 25 °C and the fermentation was continued for a total of 9 days with daily sampling to monitor OD_600_, volumetric activity towards veratryl alcohol, and protein concentration in the cell-free supernatant.

### Protein purification

The collected fermentation broth was centrifuged (11,325×*g*, 15 min, 4 °C) and the cell-free supernatant was concentrated and rebuffered in 50 mM potassium phosphate pH 6.0 using tangential flow filtration (TFF) with three membranes (10 kDa molecular cut-off, Pall, Port Washington, USA).

*Pe*AAO2 was purified by three chromatographic steps. For hydrophobic interaction chromatography (HIC), 2 M of ammonium sulfate (solid) was added to 10 ml of the concentrated supernatant and dissolved at 10 °C and rotation overnight. The sample was centrifuged (18,000×*g*, 30 min, 4 °C) and filtered using a 0.45-μm pore size filter. A XK16/20 column with Butyl Sepharose HP medium (20 ml, GE Healthcare, Chicago, USA) connected to an ÄKTApurifier FPLC-system (GE Healthcare, Chicago, USA) was equilibrated with 50 mM potassium phosphate buffer pH 6.0 with 1.5 M ammonium sulfate (eluent B). A total of 10 ml of sample was loaded onto the column and washed for two column volumes (CV) with eluent B and a flow rate of 1.5 ml/min. Proteins were eluted using a step gradient with decreasing concentrations of eluent B by mixing with 50 mM potassium phosphate buffer pH 6.0 (eluent A). Foreign proteins were removed with two CV of 70% eluent B, and PeAAO2 was eluted with three CV of 40% eluent B. Fractions showing activity towards veratryl alcohol were pooled, concentrated, and desalted using a Vivaspin Turbo 15 ultrafiltration unit (10 kDa molecular cut-off, Sartorius, Göttingen, Germany). The concentrated HIC sample was used for ion exchange chromatography (IEX) using a XK16/20 column packed with DEAE Sepharose FF medium (29 ml, GE Healthcare, Chicago, USA). The column was equilibrated with 50 mM potassium phosphate buffer pH 6.0 (eluent A) and proteins were eluted with increasing amounts of 50 mM potassium phosphate buffer pH 6.0 with 1 M sodium chloride (eluent B) at a flow rate of 1.5 ml/min. A linear gradient of 0–30% eluent B for five CV was used to elute *Pe*AAO2. Again, the active fractions were pooled and concentrated. At last, the concentrated sample was applied to a Superdex 200 Increase 10/300 GL column (24 ml, GE Healthcare, Chicago, USA) for size exclusion chromatography (SEC). Using an isocratic gradient of one CV of 50 mM potassium phosphate buffer pH 6.0 with 150 mM sodium chloride at a flow rate of 0.25 ml/min, *Pe*AAO2 was eluted and active fractions were pooled, concentrated, and desalted as described above. Purified *Pe*AAO2 was stored at 4 °C until use.

### Biochemical characterization

Protein concentration was determined by the Bradford method (Bradford 1976) with bovine serum albumin (BSA) as standard.

Glycosylation extent was analyzed by employing Peptide-N-amidase PNGase F (New England Biolabs, Frankfurt am Main, Germany) to deglycosylate 20 μg of purified *Pe*AAO2 according to the manufacturer’s protocol. The deglycosylation was carried out under denaturing as well as under native conditions (for up to 96 h) to investigate the influence of glycosylation on activity and thermal stability of *Pe*AAO2. The resulting deglycosylated protein was analyzed via SDS-polyacrylamide gel electrophoresis (SDS-PAGE). SDS-PAGE with purified enzyme samples was carried out following the protocol of Laemmli (1970) with 12.5% resolving gel. The gels were stained with Coomassie Brilliant Blue R250.

### Spectroscopic analysis

All measurements were performed at 25 °C with 2 mg/ml purified *Pe*AAO2 in 50 mM potassium phosphate buffer pH 6.0 using a Lambda 35 spectrophotometer (Perkin Elmer, Waltham, USA). The molar extinction coefficient of *Pe*AAO2 was calculated on the basis of released FAD cofactor from the purified enzyme after heat denaturation as reported elsewhere (Aliverti et al. 1999). *Pe*AAO2 was subjected to heat denaturation for 10 min at 80 °C. Precipitated protein was removed by centrifugation and the absorbance of extracted FAD was measured. The molar extinction coefficient of *Pe*AAO2 at 463 nm was calculated on the basis of the equation *ε*_463_ = *ε*_FAD_ ∗ *A*_463_/*A*_450_ with *ε*_FAD_ = 11,300 M^−1^ cm^−1^ and *A*_463_ being the absorbance of *Pe*AAO2 before heat denaturation and *A*_450_ of released FAD after heat denaturation.

### Enzymatic activity assay

The routinely used assay for determination of aryl-alcohol oxidase activity was carried out with veratryl alcohol as substrate. The measurements were conducted at room temperature in triplicates using 1-ml cuvettes with 800 μl of 100 mM sodium phosphate buffer pH 6.0 and 100 μl of 50 mM veratryl alcohol. A total of 100 μl of appropriately diluted *Pe*AAO2 in 50 mM potassium phosphate buffer pH 6.0 was added to start the reaction. Formation of veratraldehyde (*ε*_310_ = 9300 M^−1^ cm^−1^) (Guillén et al. 1992) was followed at 310 nm using an Ultrospec 7000 photometer (GE Healthcare, Chicago, USA). One unit of activity is defined as the amount of enzyme that converts 1 μmol substrate per minute under the stated conditions.

### Influence of pH and temperature on stability

Purified *Pe*AAO2 was incubated at different pH values ranging from pH 2.0 to 12.0 (at room temperature) using 100 mM Britton-Robinson buffer or at different temperatures between 4 and 80 °C in 50 mM potassium phosphate buffer at pH 6.0 for up to 1 h. Samples were taken after certain time points, incubated on ice for 5 min (in case of thermal stability) and the residual activity towards veratryl alcohol was determined. The activity assay was conducted in triplicates in microtiter plates with 20 μl of *Pe*AAO2 containing sample, 20 μl of 50 mM veratryl alcohol, and 160 μl of 100 mM sodium phosphate buffer pH 6.0. The product formation was followed at 310 nm using an Infinite M200 Pro plate reader (Tecan, Männedorf, Switzerland). For determination of *T*_50_, the temperature at which the enzyme loses 50% of activity, *Pe*AAO2 was incubated at temperatures ranging from 45 to 75 °C for 10 min. Afterwards, the samples were cooled on ice for 10 min before measuring the residual activity towards veratryl alcohol as stated above. The resulting data set was plotted using the program OriginPro 9.0 (OriginLab Corporation, Northampton, MA, USA) and the *T*_50_ value was determined by fitting the data using the Boltzmann equation.

### Determination of melting temperature

To identify the melting temperature (*T*_M_) of purified and of natively *N*-deglycosylated *Pe*AAO2, the change of intrinsic FAD cofactor fluorescence was monitored in dependence of temperature as employed in the *Thermo*FAD assay (Forneris et al. 2009). *Pe*AAO2 was diluted in 50 mM potassium phosphate buffer pH 6.0 to 1 mg/ml and 25 μl of diluted sample (in triplicate) was used to monitor the fluorescence at different temperatures using qPCR cycler qTOWER^3^ (Analytik Jena, Jena, Germany). Excitation wavelength was set to 470 nm and emission wavelength to 520 nm using the SYBR Green fluorescence filter. A temperature gradient from 15 to 95 °C in 0.5 °C increments after 15 s delay was used. The first derivative of the melting curve was calculated using the program OriginPro 9.0 and the *T*_M_ value was extracted as maximum of the first derivative.

### Investigation of substrate spectrum

Activity of *Pe*AAO2 towards 41 compounds was tested in a coupled assay making use of the generated hydrogen peroxide as product of AAO activity. The coupled system included horseradish peroxidase (HRP, Type VI, Sigma-Aldrich, Schnelldorf, Germany) and 2,2′-azino-bis(3-ethylbenzothiazoline-6-sulphonic acid) (ABTS). The measurements were conducted in triplicates in 96-well plates in a total volume of 200 μl at room temperature. For this, 20 μl of a suitable *Pe*AAO2 dilution was mixed with 20 μl of 10 mM substrate (with residual percentage of appropriate organic solvent, see Supplemental Table S1), 20 μl of 50 mM ABTS, 20 μl of 1 mg/ml HRP, and 120 μl of 100 mM potassium phosphate buffer pH 6.0. Oxidation of ABTS by HRP in the presence of hydrogen peroxide was followed spectrophotometrically at 420 nm for 3 m (*ε*_420_ = 36,000 M^−1^cm^−1^) (Childs and Bardsley 1975) using an Infinite M200 Pro plate reader (Tecan, Männedorf, Switzerland).

### Determination of kinetic constants

Kinetic constants *V*_max_ and *K*_M_ were determined for selected substrates at varying concentrations at 25 °C in 100 mM sodium phosphate buffer pH 6.0 in triplicates in a UV-Star® 96-well micro titer plate (Greiner Bio-One GmbH, Frickenhausen, Germany) with 200 μl assay volume using an Infinite M200 Pro plate reader (Tecan, Männedorf, Switzerland). The tested substrates were *p*-anisyl alcohol (0.98 μM to 1 mM), benzyl alcohol (9.8 μM to 10 mM), cinnamyl alcohol (9.8 μM to 20 mM in DMSO), cumic alcohol (9.8 μM to 10 mM), *trans,trans*-2,4-hexadienol (0.98 μM to 1.75 mM), piperonyl alcohol (0.98 μM to 1 mM), and veratryl alcohol (9.8 μM to 10 mM). The molar extinction coefficients used for calculation were *p*-anisaldehyde *ε*_285_ = 16,980 M^−1^ cm^−1^ (Guillén et al. 1992), benzaldehyde *ε*_250_ = 13,800 M^−1^ cm^−1^ (Guillén et al. 1992), cinnamaldehyde *ε*_310_ = 15,600 M^−1^ cm^−1^ (Ferreira et al. 2005), veratraldehyde *ε*_310_ = 9300 M^−1^ cm^−1^, and *trans,trans*-2,4-hexadienal *ε*_280_ = 30,140 M^−1^ cm^−1^ (Ruiz-Dueñas et al. 2006). The molar extinction coefficients of cuminaldehyde (*ε*_262_ = 2920 M^−1^cm^−1^) and piperonal (*ε*_317_ = 8680 M^−1^cm^−1^) were determined as shown in the Supplemental Figs. S1, S2, S3 and S4. Results were analyzed using OriginPro 9.0. A non-linear regression using the Michaelis-Menten equation was conducted to yield the maximum rate *V*_max_ and the Michaelis constant *K*_M_, and led to the calculation of the rate constant *k*_cat_ and catalytic efficiency *k*_cat_/*K*_M_ based on the molar concentration as determined by using the calculated molar extinction coefficient of *Pe*AAO2.

## Results

### Enzyme production and purification

**Fig. 1:**
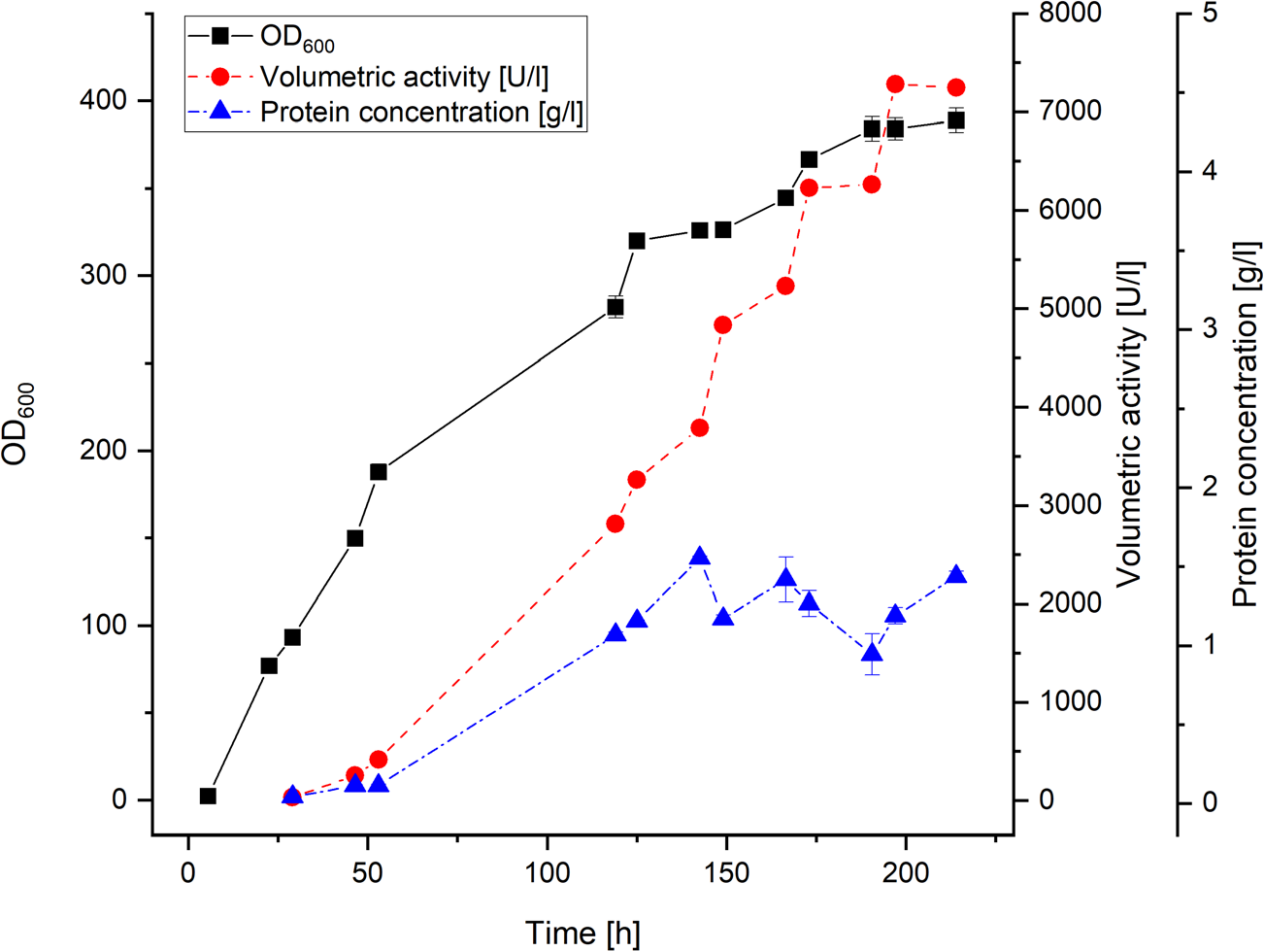
Fed-batch cultivation of recombinant *P. pastoris* X-33 in a 7.5-l bioreactor to produce *Pe*AAO2. Squares: OD_600_ values; circles: volumetric activity (U/l) in cell-free supernatant; triangles: protein concentration (g/l). All measurements were done in triplicate

The *P. pastoris* (*K. phaffii*) expression vector pPICZA harboring the codon-optimized *peaao2* gene with its native signal sequence under control of the methanol inducible P_AOX1_ promoter was integrated into the genome of *P. pastoris* X-33 by homologous recombination. Six *P. pastoris* transformants were screened for secretion of active *Pe*AAO2 in BMMY medium in shaking flasks. After 2 days of expression, the volumetric activities towards veratryl alcohol ranged from 18.4 to 74.0 U/l for different transformants. The *P. pastoris* transformant with the highest volumetric activity was subsequently used for enzyme production in a 7.5-l bioreactor. After 9 days of fed-batch cultivation, the OD_600_ of the culture reached its maximum at 389 accompanied by a volumetric activity of 7250 U/l at a protein concentration of 1.4 g/l (Fig. 1).

After cell separation and supernatant concentration by tangential flow filtration (TFF), recombinant *Pe*AAO2 was purified to homogeneity in a three-step purification procedure, including hydrophobic interaction (HIC), ion exchange (IEX), and size exclusion chromatography (SEC) (Table 1). The purified enzyme showed a specific activity of 23.0 U/mg towards veratryl alcohol, and was strongly yellow in color and slightly viscous. The expression yield calculated on the basis of specific activity of *Pe*AAO2 was 315 mg/l of culture.

**Table 1:** Purification of recombinant *Pe*AAO2

Purification step	Total protein (mg)^c^	Total activity (U)^d^	Specific activity (U/mg)	Yield (%)^e^	Purity (x-fold)
Supernatant^a^	5030	25,400	5.0	–	1.0
TFF 1st eluate^b^	860	10,200	11.8	100	2.3
Butyl Sepharose HP	50	648	13.0	64	2.6
DEAE Sepharose HP	26.4	439	16.6	43	3.3
Superdex 200 increase	12.6	291	23.0	29	4.6

^a^Cell-free supernatant after centrifugation of fermentation broth

^b^Ultrafiltration retentate of supernatant using tangential flow filtration (TFF). Concentrated sample was collected in three steps (eluates) with different enzyme activities and protein concentrations. Only the first eluate was used for chromatographic purifications. Hence the apparent loss of activity

^c^Protein concentration was estimated by Bradford assay with BSA as standard

^d^Enzyme activity was measured with veratryl alcohol

^e^Yield based on 10 ml of the 1st eluate applied to Butyl Sepharose HP

**Fig. 2:**
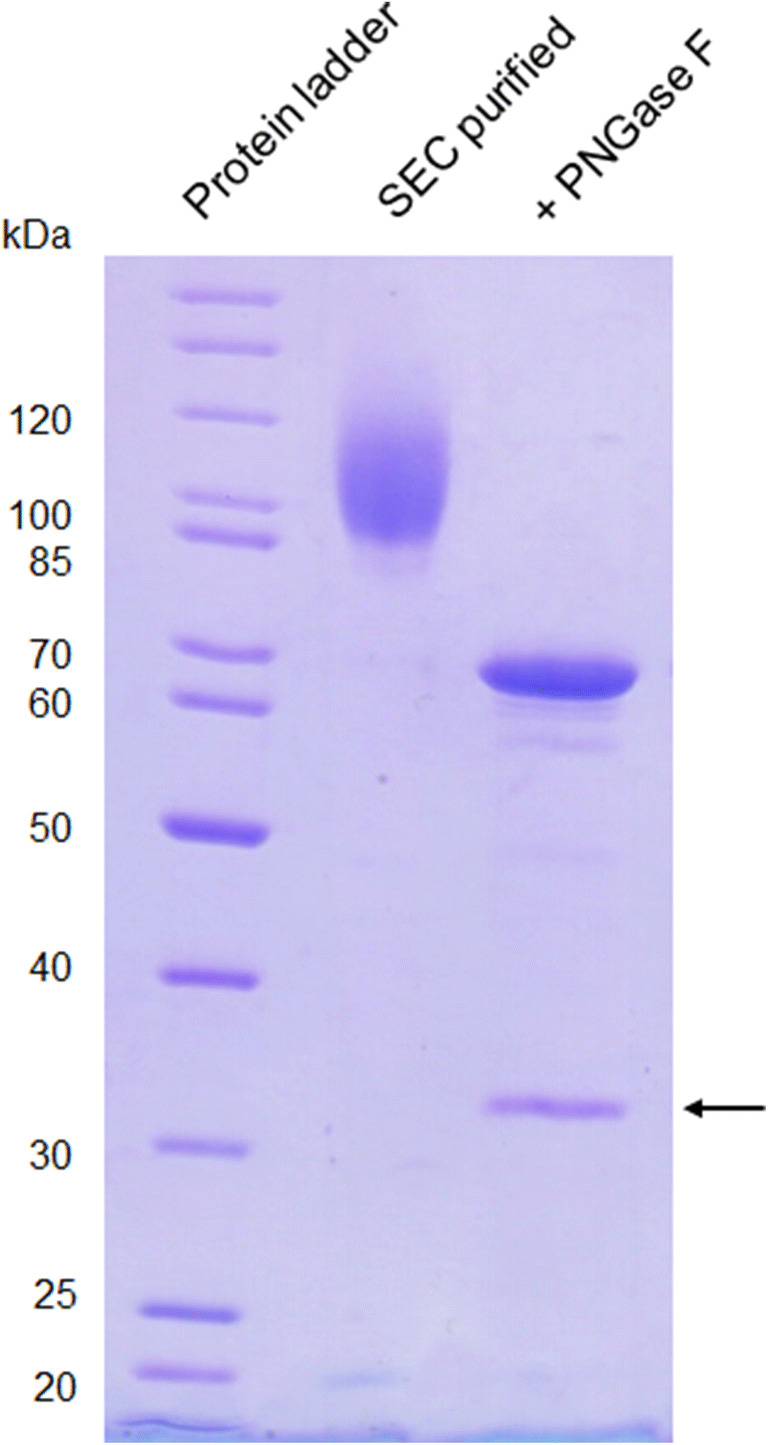
SDS-PAGE analysis of purified *Pe*AAO2 and PNGase F treated *Pe*AAO2. A total of 5 μg of each sample was loaded and separated in a 12.5% resolving gel. Arrow indicates PNGase F (36 kDa)

Native PAGE demonstrated that purified *Pe*AAO2 is present in solution as monomer (Supplemental Fig. S5). SDS-PAGE analysis of purified *Pe*AAO2 revealed a strong band at around 100 kDa (Fig. 2). The theoretical molecular weight of *Pe*AAO2 without signal peptide (first 27 amino acids; the same signal peptide as of the closely related *Pe*AAO, Varela et al. 1999) was predicted to be 60.8 kDa (using Protparam ExPAsy) (Gasteiger et al. 2005). After *N*-deglycosylation using PNGase F, a shift of mobility to around 70 kDa was observed, indicating at least 30% *N*-glycosylation of heterologously expressed *Pe*AAO2 (Fig. 2).

**Fig. 3:**
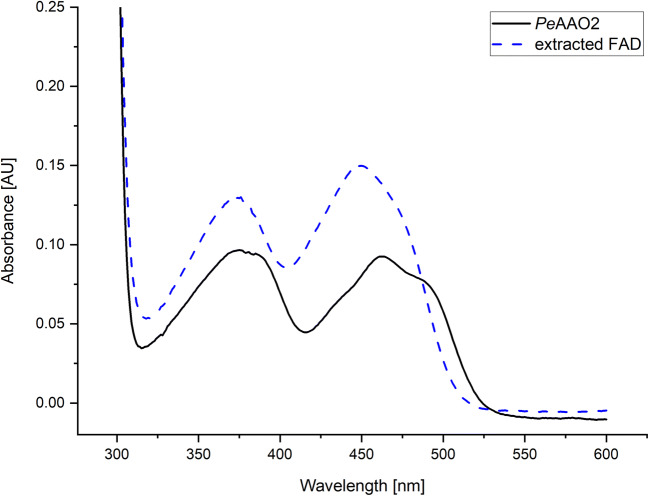
UV-Vis spectrum of purified *Pe*AAO2. Solid line: native *Pe*AAO2 in its oxidized form; dashed line: extracted FAD after heat denaturation

The purified *Pe*AAO2 was analyzed in terms of its spectroscopic properties (Fig. 3). The oxidized enzyme showed two maxima at 376 nm and 463 nm. The extracted FAD showed two pronounced maxima at 376 nm and 450 nm. The estimated molar extinction coefficient of *Pe*AAO2 at 463 nm (*ε*_463_ ) was 7029 M^−1^ cm^−1^.

### Influence of pH, temperature, and glycosylation on enzyme stability

**Fig. 4:**
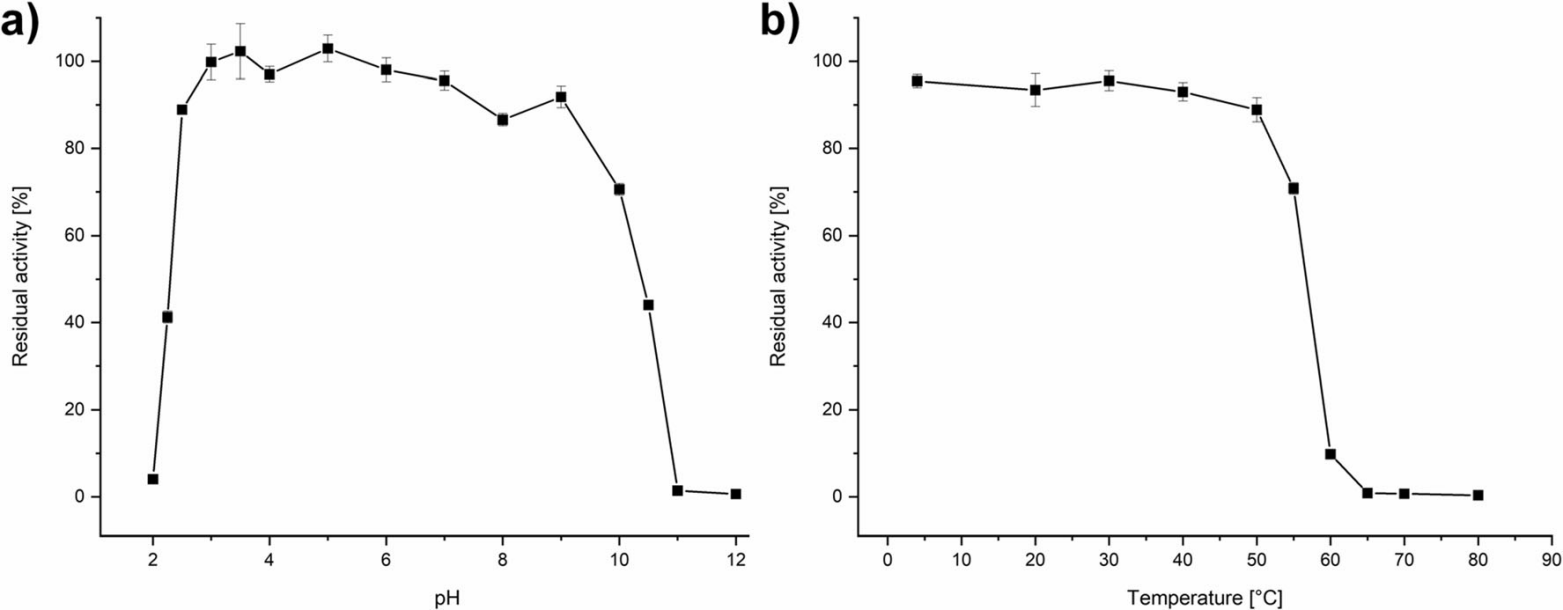
Influence of pH and temperature on stability of *Pe*AAO2. **a** pH stability was determined in 100 mM Britton-Robinson buffer at the corresponding pH for 1 h at room temperature. **b** Thermal stability was investigated from 4 to 80 °C in 50 mM potassium phosphate buffer pH 6.0 for 1 h. Residual activity is given in % of initial activity without incubation

pH stability of *Pe*AAO2 was investigated at various pH values between 2.0 and 12.0 and the enzyme remained stable over a wide range from pH 3.0 to 9.0 with residual activities of around 90% after 1 h incubation at room temperature (Fig. 4a), while a total loss of activity at pH 2.0 and pH 11.0 after 1 h incubation was observed. Thermal stability of *Pe*AAO2 was studied at temperatures between 4 and 80 °C for up to 1 h incubation at pH 6.0. *Pe*AAO2 was stable from 4 to 50 °C with residual activities of around 90%, while residual activity dropped to 70% and 10% after 1 h of incubation at 55 °C and 60 °C, respectively (Fig. 4b).

The temperatures at which half of the activity of *Pe*AAO2 was lost after 10 min of incubation (*T*_50_) and the melting temperature (*T*_M_) of *Pe*AAO2 were determined as well. *Pe*AAO2 showed a *T*_50_ value of 62.1 °C, while the *T*_M_ value was 65.5 °C. For natively *N*-deglycosylated *Pe*AAO2, a *T*_M_ value of 57.0 °C was measured. The deglycosylated enzyme showed a residual activity of 98.5% as compared with *Pe*AAO2 incubated under the same conditions but without PNGase F.

### Substrate spectrum

A coupled colorimetric assay using ABTS and HRP to measure hydrogen peroxide produced by AAO upon substrate oxidation was used to determine the substrate spectrum of *Pe*AAO2. A total of 41 compounds, some of which have been described as aryl-alcohol oxidase substrates including benzylic, other cyclic, heterocyclic, and aliphatic alcohols, were investigated (Table 2). The activity towards benzyl alcohol was set to 100%. Benzylic alcohols methoxylated in *para*-position like *p*-anisyl alcohol (647%), veratryl alcohol (322%), and isovanillyl alcohol (246%) were much better substrates than benzyl alcohol. The presence of an extended unsaturated side chain as in cinnamyl alcohol increased activity as well (442%). The enzyme showed the highest relative activity of 874% towards bicyclic 2-naphthalenemethanol, followed by the aliphatic and unsaturated *trans,trans*-2,4-hexadienol and *trans,trans*-2,4-heptadienol with 807% and 737%, respectively. Also, the heterocyclic benzodioxol derivative piperonyl alcohol was accepted by *Pe*AAO2 and oxidized with a relative activity of 301%, while with the isopropyl substituted benzylic alcohol - cumic alcohol, a relative activity of 149% was reached. All other tested compounds were “worse” substrates for *Pe*AAO2 and led to lower relative activities compared to benzyl alcohol. Amino substituted 3- and 4-aminobenzyl alcohols were oxidized with relative activities of 9.4 and 18.6%, respectively, while nitrogen-containing heterocyclic compounds like pyridine and indole derivatives were converted with relative activities of 2% or below. Among the unsaturated aliphatic alcohols, *trans*-2-hexenol (64%), *trans*-2-heptenol (32%), *trans*-2-octenol (5.2%), and *trans*-2-*cis*-6-nonadienol (3.3%) were oxidized. The investigated branched aliphatic alcohols were only accepted to a very small extent as compared with benzyl alcohol, with relative activities generally below 5%.

**Table 2:**
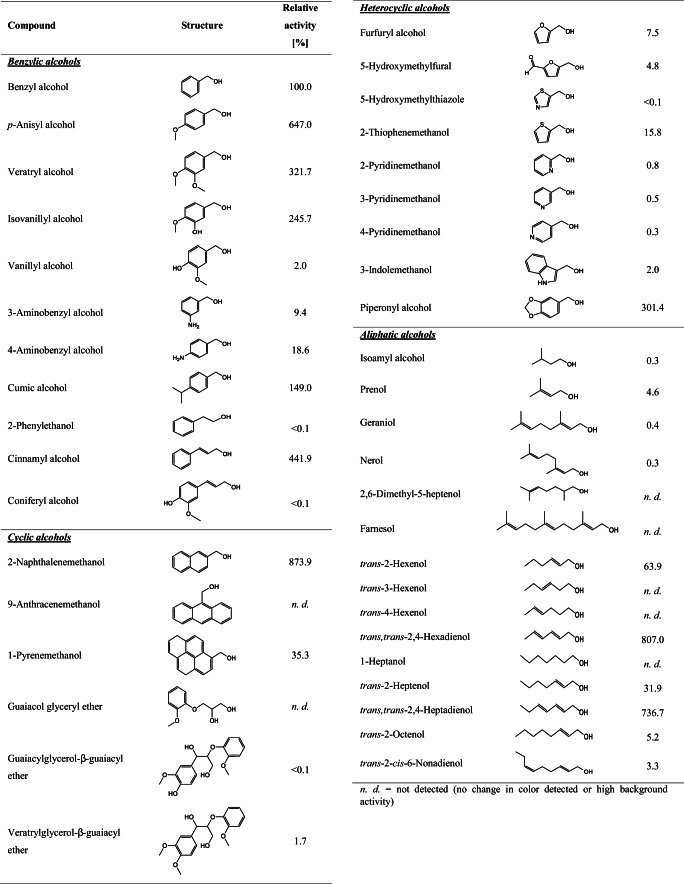
Substrate scope of *Pe*AAO2. Generated H_2_O_2_ formed upon substrate oxidation was detected in a coupled ABTS-HRP assay. Activity towards benzyl alcohol was set to 100%

### Kinetic constants

Kinetic constants *K*_M_, *k*_cat_, and *k*_cat_/*K*_M_ of *Pe*AAO2 for some of the substrates identified during substrate screening were determined at pH 6.0 (Table 3), at which *Pe*AAO2 showed the highest activity (Supplemental Fig. S6). *Pe*AAO2 showed the highest affinity (*K*_M_) towards *p*-anisyl alcohol with 24.3 μM followed by piperonyl alcohol with 59.1 μM and the lowest affinity was found for cinnamyl alcohol with 1912 μM. The highest catalytic efficiencies (*k*_cat_/*K*_M_) with 2436 mM^−1^ s^−1^ and 600.2 mM^−1^ s^−1^ were also estimated for *p*-anisyl alcohol and piperonyl alcohol. Using cumic alcohol as substrate, the highest turnover rate (*k*_cat_) was observed with 160.8 s^−1^, which is 4-fold higher than for benzyl alcohol.

**Table 3:** Kinetic constants of *Pe*AAO2 compared with those of other AAOs

		*Pe*AAO2 from *P. eryngii* expressed in *P. pastoris*^a^	*Pe*AAO from *P. eryngii* expressed in *A. nidulans*^*b*^	*Pe*AAO FX9 variant from *P. eryngii* expressed in *P. pastoris*^c^
*p*-Anisyl alcohol	*K*_M_ (μM)	24.3 ± 0.8	27	37
	*k*_cat_ (s^−1^)	59.2 ± 0.04	142	70
	*k*_cat_/*K*_M_ (mM^−1^ s^−1^)	2436	5230	1909
Benzyl alcohol	*K*_M_ (μM)	599.6 ± 18.7	632	440
	*k*_cat_ (s^−1^)	12.8 ± 0.01	30	34
	*k*_cat_/*K*_M_ (mM^−1^ s^−1^)	21.39	47	78
Cinnamyl alcohol	*K*_M_ (μM)	2740 ± 103	*n.d.*	*n.d.*
	*k*_cat_ (s^−1^)	125.5 ± 0.1	*n.d.*	*n.d.*
	*k*_cat_/*K*_M_ (mM^−1^ s^−1^)	45.80	*n.d.*	*n.d.*
Cumic alcohol	*K*_M_ (μM)	1912 ± 42.4	*n.d.*	*n.d.*
	*k*_cat_ (s^−1^)	160.8 ± 0.1	*n.d.*	*n.d.*
	*k*_cat_/*K*_M_ (mM^−1^ s^−1^)	84.1	*n.d.*	*n.d.*
*trans,trans*-2,4-hexadienol	*K*_M_ (μM)	143.6 ± 11.5	94	106
	*k*_cat_ (s^−1^)	68.8 ± 0.05	119	89
	*k*_cat_/*K*_M_ (mM^−1^ s^−1^)	479.3	1270	840
Piperonyl alcohol	*K*_M_ (μM)	59.1 ± 3.0	*n.d.*	*n.d.*
	*k*_cat_ (s^−1^)	35.5 ± 0.02	*n.d.*	*n.d.*
	*k*_cat_/*K*_M_ (mM^−1^ s^−1^)	600.2	*n.d.*	*n.d.*
Veratryl alcohol	*K*_M_ (μM)	446.6 ± 7.5	540	410
	*k*_cat_ (s^−1^)	47.2 ± 0.03	114	57
	*k*_cat_/*K*_M_ (mM^−1^ s^−1^)	105.7	210	139

*n.d.* not determined

^a^(This study), 100 mM sodium phosphate buffer pH 6.0. 25 °C, all measurements in triplicate

^b^(Ferreira et al. 2006), 100 mM sodium phosphate buffer pH 6.0, 24 °C

^c^(Viña-Gonzalez et al. 2018), 100 mM sodium phosphate buffer pH 6.0, 25 °C

## Discussion

### Enzyme production and properties

The efficient utilization of AAOs in biocatalytic processes is mainly hampered due to the lack of high-yield expression systems. Our attempts to express the well-examined *Pe*AAO from *P. eryngii* ATCC 90787 in *P. pastoris* led to no detectable activity (unpublished data), while the expression level of *Pe*AAO2 from *P. eryngii P34* reached 315 mg/l and exceeded that of the in *P. pastoris* expressible and “engineered” variant *Pe*AAO FX9 with 25.5 mg/l by factor 12 (Viña-Gonzalez et al. 2018). Thus, *Pe*AAO2 is the best expressed *Pleurotus* AAO in *P. pastoris* described so far. *Pe*AAO2 and *Pe*AAO differ in seven amino acid positions located on or near the surface of the protein (Supplemental Fig. S7 and S8). The active site including the two catalytic active histidine residues (His529 and His573 (Ferreira et al. 2006)) and the hydrophobic substrate access channel (Tyr119, Phe424, and Phe528 (Fernández et al. 2009)) are identical in both enzymes, but an additional potential *N*-glycosylation site (Asn361-X-Ser) is present in *Pe*AAO2. Which of the amino acid variations leads to (high) expression of *Pe*AAO2 in *P. pastoris* as compared with *Pe*AAO remains questionable and is under further investigation.

*Pe*AAO2 contains eight potential *N*-glycosylation sites (Asn-X-Thr/Ser, where X is any amino acid except for proline) (Kukuruzinska et al. 1987) at the residues Asn89, Asn165, Asn178, Asn249, Asn336, Asn352, Asn361, and Asn396 (Supplemental Fig. S7). The discrepancy in molecular weight of *Pe*AAO2 with a theoretical molecular weight without signal peptide of 60.8 kDa and 100 kDa observed via SDS-PAGE is due to *N*- and *O*-glycosylation performed by *P. pastoris*. The *N*-deglycosylated enzyme showed a sharp band at 70 kDa, indicating 30% of *N*-glycosylation extent in recombinantly produced *Pe*AAO2, while 10% *O*-glycosylation is assumed. This value is higher than the carbohydrate content described for homologously produced *Pe*AAO with 14% (Varela et al. 2000b). Interestingly, the *Pe*AAO variant FX9 expressed in *P. pastoris* was poorly glycosylated, despite the presence of seven potential *N*-glycosylation sites (Viña-Gonzalez et al. 2018).

The *N*-deglycosylated *Pe*AAO2 retained its activity after deglycosylation, implying that glycosylation is not necessary for enzymatic activity, but rather positively affects enzyme thermostability. The glycosylated *Pe*AAO2 showed a 9 °C higher *T*_M_ value than the *N*-deglycosylated enzyme, which confirms that glycosylation enhances thermostability (Wang et al. 1996). Indeed, the glycosylated *Pe*AAO2 exhibited 90% of residual activity after 1 h incubation at 50 °C and showed a *T*_50_ value of 62.1 °C, which is comparable with that of hyperglycosylated *Pe*AAO variant FX9 expressed in *S. cerevisiae* (63.0 °C) (Viña-Gonzalez et al. 2018). In contrast, *Pe*AAO purified from inclusion bodies from *E. coli* lacks glycosylation and shows a much lower thermostability compared to *Pe*AAO2 and *Pe*AAO variant FX9 with around 20% of residual activity after 50 min incubation at 50 °C and a *T*_50_ of 47.5 °C (Ruiz-Dueñas et al. 2006; Viña-Gonzalez et al. 2015). *Pe*AAO2 showed high stability within a wide pH range between pH 3.0 and 9.0, which is similar to another glycosylated *Pleurotus* AAO (Viña-Gonzalez et al. 2015). Since the *E. coli*-derived *Pe*AAO showed considerably lower pH stability, especially at pH 3.0 and above pH 9.0 (Viña-Gonzalez et al. 2015), we assume that high pH stability of AAOs is also attributed to glycosylation.

### Substrate scope of *Pe*AAO2

*Pe*AAO2 was found to oxidize a broad range of chemically diverse primary alcohols, including compounds not yet reported as substrates for AAOs. The substrate preference was dependent on the present aromatic substitution groups and the number of conjugated double bonds, as reported also for other *Pleurotus* AAOs (Bourbonnais and Paice 1988; Guillén et al. 1992). A methoxy-group at the *para*-position of the aromatic ring seems to be crucial for efficient substrate oxidation as shown for *p*-anisyl alcohol, veratryl alcohol, and isovanillyl alcohol when compared with the non-substituted benzyl alcohol. A *para*-isopropyl group in cumic alcohol had also a beneficial effect leading to a 1.5-times higher relative activity than with benzyl alcohol. These results allow to suggest that the presence of an electron-donating group at *para*-position had a positive effect on enzyme activity. Presumably, enhanced electron density at the aromatic ring facilitates oxidation of the primary alcohol group. The presence of an amino group in *para*-position in 4-aminobenzyl alcohol reduced substrate acceptance by a factor of 5, which might be explained by its protonated state at neutral pH, which makes this group electron-withdrawing. The presence of unsaturated bonds in the side chains of (aryl) alkyl alcohols, and thus extension of the conjugated double bond system as in cinnamyl alcohol had tremendous effects on substrate oxidation resulting in 4-times higher relative activity compared to benzyl alcohol. Although coniferyl alcohol contains an unsaturated side chain, its oxidation was barely detectable as was also seen for the *meta*-methoxy-*para*-hydroxy substituted vanillyl alcohol. In both substrates, a methoxy-group at *meta*-position of the aromatic ring act as electron-withdrawing group. Interestingly, the presence of a *para*-hydroxyl group has been previously reported to negatively influence the oxidation reaction by an AAO (Guillén et al. 1992). Obviously, the influence of the substrate binding site on substrate specificity and enzyme activity should be considered as well.

Expansion of the aromatic system to two condensed aromatic rings in 2-naphthalenemethanol led to the highest activity detected among all substrates, which is in accordance with activity of *Pe*AAO (Guillén et al. 1992). However, further extension of the ring system as in the tricyclic 9-anthracenemethanol did not result in detectable substrate oxidation, while the four-membered ring of 1-pyrenemethanol was oxidized with one third of activity as compared with benzyl alcohol. The acceptance of 9-anthracenemethanol by *Pe*AAO could be affected by steric hindrances: The primary alcohol group is located in a less exposed position as compared with 2-naphthalenemethanol or 1-pyrenemethanol, thereby not reaching into the active site cavity.

Various heterocyclic compounds derived from benzodioxole, furan, indole, pyridine, and thiophene were tested as substrates and were accepted by *Pe*AAO2 at least to some extent. Most remarkably, the benzodioxole derivative piperonyl alcohol was oxidized with a 3-times higher relative activity than benzyl alcohol. As described for veratryl alcohol (Guillén et al. 1992), the oxygen atoms in piperonyl alcohol most likely produce an electron-donating effect, leading to a higher electron density at the primary hydroxyl group, resulting in favored oxidation of the primary alcohol group. *Pe*AAO2’s activity was rather low towards the furan-derived 5-hydroxymethylfurfural (5-HMF) which has been investigated in several studies as starting material for the production of bio-based 2,5-furandicarboxylic acid (FDCA) as precursor for plastics with involvement of an AAO (Carro et al. 2014; Karich et al. 2018; Serrano et al. 2019a; Viña-Gonzalez et al. 2020). The sulfur-containing 2-thiophenemethanol was converted with a relative activity of 16% and was the first described sulfuric compound accepted by an AAO. Several nitrogen-containing heterocyclic compounds including indole and pyridine derivatives were converted by *Pe*AAO2 only to less than 2%. Even though the substrate oxidation was rather low with some of these heterocyclic compounds, the results show that *Pe*AAO2 is capable of oxidizing chemically diverse primary alcohols.

Linear primary alcohols can serve as AAO substrates, if the alcohol group is in conjugation with double bonds, like in *trans,trans*-2,4-hexadienol (Guillén et al. 1992) or *trans,trans*-2,4-heptadienol, which led to the second and third highest relative activity of all tested compounds. The reduction of number of conjugated double bonds as in *trans*-2-hexenol and *trans*-2-heptenol resulted in 12-fold and 23-fold lower relative activity than with their counterparts *trans,trans*-2,4-hexadienol and *trans,trans*-2,4-heptadienol. The elongation of the linear unsaturated alcohol to C8 and C9 as in *trans*-2-octenol and *trans*-2-*cis*-6-nonadienol reduced enzyme activity further. Nevertheless, oxidation of the latter substrate results in “the violet leaf aldehyde” or “cucumber aldehyde,” which is the major aroma component in fresh cucumber (Schieberle et al. 1990) and among the most potent fragrance compounds (Surburg and Panten 2006). This volatile compound is also present in different plant materials including extracts of violet leafs and fruits such as cherry and mango (Pino and Mesa 2006; Schmid and Grosch 1986). Several other aliphatic alcohols like geraniol, nerol, and prenol were accepted by *Pe*AAO2 and converted with activities below 5%, possibly due to steric limitations caused by their branched aliphatic structure.

Substrate affinities of *Pe*AAO2 for *p*-anisyl alcohol, benzyl alcohol, *trans,trans*-2,4-hexadienol, and veratryl alcohol were in the same range as for the closely related *P. eryngii Pe*AAO expressed in *A. nidulans* and its FX9 variant expressed in *P. pastoris* (Table 2). All three enzymes showed the highest catalytic efficiency for *p*-anisyl alcohol. Glycosylated wild-type *Pe*AAO with 14% carbohydrate content, expressed in *A. nidulans* (Varela et al. 2001), showed higher catalytic efficiencies for most substrates compared to *Pe*AAO2 with 30% carbohydrate content and the poorly glycosylated *Pe*AAO variant FX9 (Viña-Gonzalez et al. 2018). Lower activity of variant FX9 compared to *Pe*AAO might be caused by introduced mutations. On the other hand, it has been shown that non-glycosylated *Pe*AAO derived from *E. coli* showed lower catalytic efficiencies than glycosylated *Pe*AAO (Ruiz-Dueñas et al. 2006). Besides having a positive effect on pH and thermal stability, catalytic efficiency of AAOs seems to be positively influenced by glycosylation as well. Other AAOs that have been expressed in *P. pastoris* include *Coprinopsis cinerea* (*Cc*AAO) (Tamaru et al. 2018) and *Ustilago maydis* AAO (*Um*AAO) (Couturier et al. 2016). *Um*AAO exhibited the highest catalytic efficiency towards *p*-anisyl alcohol similar to *Pleurotus* AAOs, while for *Cc*AAO, the highest catalytic efficiency was described for benzyl alcohol. These results indicate different substrate specificities among different fungal AAOs.

As far as to our knowledge, the acceptance of piperonyl alcohol and cumic alcohol, as well as of amino-substituted and thiophene-derived primary alcohols and 1-pyrenemethanol, has not been reported for AAOs so far, and extends our knowledge of the substrate scope of aryl-alcohol oxidases. The second highest catalytic efficiency of *Pe*AAO2 was observed with piperonyl alcohol, proving that this compound is a promising substance for biocatalytic conversions, as its aldehyde is the fragrance compound piperonal used in cosmetics, and flavor and fragrance industry. Piperonal has a sweet-flowery and spicy odor and is present in essential oils of flowers of the *Heliotropium* genus (Bellardita et al. 2014; Santos et al. 2003) and thus also termed as “heliotropin”. Due to its benzodioxole functionality, it also serves as intermediate for several products of industrial importance, such as insecticides, pesticides, and pharmaceutical products, e.g., used in the synthesis of new drugs against Alzheimer’s disease (Brum et al. 2019; Santos et al. 2004; Wang et al. 2019).

The highest turnover rate for *Pe*AAO2 was observed for cumic alcohol oxidation to the bioactive compound cuminaldehyde, a major constituent of seed oil of *Cuminum cyminum* plant (Lee 2005; Li and Jiang 2004). Beyond the use of *C. cyminum* seeds as spice in traditional cuisines, different beneficial effects have been attributed to its use, including anticancer, antidiabetic, and neuroprotective effects that have been linked to cuminaldehyde as its active ingredient (Lee 2005; Morshedi et al. 2015; Patil et al. 2013; Tsai et al. 2016). The biocatalytic production of cuminaldehyde has not been described yet and the oxidation of cumic alcohol to cuminaldehyde using *Pe*AAO2 seems to be a feasible route.

In summary, high-yield production of *Pe*AAO2 in *P. pastoris* together with its broad substrate spectrum and high stability renders this enzyme a promising candidate for biotechnological applications. Additionally, the production of piperonal and cuminaldehyde by *Pe*AAO2 further expands the use of this biocatalyst for the production of intermediates for pharmaceutical products, as well as of flavors and fragrances.

## Electronic supplementary material

ESM 1 (PDF 910 kb)

## Data Availability

All data on which the conclusions were drawn are presented in this study.
