# Supplementary material for: High-level expression of aryl-alcohol oxidase 2 from Pleurotus eryngii in Pichia pastoris for production of fragrances and bioactive precursors
Source: Appl Microbiol Biotechnol. 2020 Sep 19;104(21):9205–18. doi: 10.1007/s00253-020-10878-4 (PMC7567689; doi:10.1007/s00253-020-10878-4)
Supplement: Supplementary file 1 — (PDF 910 kb) [file 253_2020_10878_MOESM1_ESM.pdf]

ELECTRONIC SUPPLEMENTARY MATERIAL

High-level expression of aryl-alcohol oxidase 2 from *Pleurotus eryngii* in *Pichia pastoris* for production of fragrances and bioactive precursors

Nina Jankowski [1], Katja Koschorreck[1], Vlada B. Urlacher [1#]

[1] Institute of Biochemistry at Heinrich-Heine-University Düsseldorf, Universitätsstraße 1, 40225 Düsseldorf, Germany

[#]Address correspondence to

Vlada B. Urlacher

vlada.urlacher@uni-duesseldorf.de

Supplementary Materials and Methods

Compounds and solvents used in substrate screening

In total, 41 different benzylic, cyclic, heterocyclic and aliphatic alcohols were used at 1 mM final concentration in the coupled ABTS-HRP assay. The solutions of tested compounds were prepared using the following solvents (Table S1).

Table S1 Compounds used in substrate screening

	**Substrate**	**Manufacturer**	**Solvent for 10 mM Stock**
**Benzylic alcohols**	3-Aminobenzyl alcohol	TCI	ddH_2O
	4-Aminobenzyl alcohol	TCI	10 % DMSO
	p-Anisyl alcohol	Sigma-Aldrich	ddH_2O
	Benzyl alcohol	AppliChem	ddH_2O
	Cinnamyl alcohol	Acros Organics	10 % DMSO
	Coniferyl alcohol	Sigma-Aldrich	10 % DMSO
	Cumic alcohol	Acros Organics	10 % DMSO
	Isovanillyl alcohol	BLDpharm	10 % DMSO
	2-Phenylethanol	Carl Roth	10 % DMSO
	Vanillyl alcohol	Acros Organics	10 % DMSO
	Veratryl alcohol	Acros Organics	ddH_2O
**Cyclic alcohols**	9-Anthracenemethanol	BLDpharm	100 % DMSO *
	2-Naphthalenemethanol	Acros Organics	10 % DMSO
	1-Pyrenemethanol	TCI	100 % DMSO *
	Guaiacol glyceryl ether	Sigma-Aldrich	ddH_2O
	Guaiacylglycerol-β-guaiacyl ether	abcr	10 % DMSO
	Veratrylglycerol-β-guaiacyl ether	abcr	10 % DMSO
**Heterocyclic alcoshols**	Furfuryl alcohol	Sigma-Aldrich	ddH_2O
	5-Hydroxymethylfural	Carbolutions	ddH_2O
	5-Hydroxymethylthiazole	J&K	10 % DMSO
	3-Indolemethanol	J&K	100 % DMSO
	Piperonyl alcohol	J&K	10 % DMSO
	2-Pyridinemethanol	J&K	ddH_2O
	3-Pyridinemethanol	J&K	ddH_2O
	4-Pyridinemethanol	Acros Organics	ddH_2O
	2-Thiophenemethanol	Sigma-Aldrich	10 % DMSO
**Aliphatic alcohols**	Farnesol	Acros Organics	10 % DMSO
	Geraniol	Sigma-Aldrich	100 % ethanol

2,6-Dimethyl-5-heptenol	Sigma-Aldrich	10 % DMSO
1-Heptanol	Acros Organics	10 % DMSO
trans-2-Heptenol	TCI	10 % DMSO
trans,trans-2,4-Heptadienol	Alfa Aesar	10 % DMSO
trans-2-Hexenol	Alfa Aesar	10 % DMSO
trans-3-Hexenol	Alfa Aesar	10 % DMSO
trans-4-Hexenol	Fluorochem	10 % DMSO
trans,trans-2,4-Hexadienol	Acros Organics	ddH_2O
Isoamyl alcohol	TCI	100 % ethanol
Nerol	Sigma-Aldrich	100 % ethanol
trans-2-*cis*-6-Nonadienol	Sigma-Aldrich	10 % DMSO
trans-2-Octenol	Alfa Aesar	10 % DMSO
Prenol	TCI	10 % DMSO

* for 9-anthracenemethanol and 1-pyrenemethanol the final DMSO concentration was 20 % in the assay mixture as additional 10 % of DMSO was added to the 10 % from 10 mM stock (in 100 % DMSO) solutions for final concentration of 1 mM

Determination of molar extinction coefficients for cuminaldehyde and piperonal

Cumic alcohol (Acros Organics), cuminaldehyde (J&K Scientific), piperonyl alcohol (J&K Scientific) and piperonal (Sigma-Aldrich) were prepared as stock solutions at 10 mM in 100 mM sodium phosphate buffer pH 6.0 and dissolved by gentle shaking and warming of the solutions. For recording of the spectra, all samples were diluted in the same buffer as mentioned. The spectra were recorded from 200 to 400 nm in a Quartz cuvette using a Lambda 35 spectrophotometer (Perkin Elmer, Waltham, USA) at 25 °C. The absorption maxima of alcohol and aldehyde were extracted from the obtained spectra.

For measurements of absorbance at the corresponding maxima of the aldehydes, further dilutions were prepared: Cuminaldehyde dilutions ranged from 0.2 mM to 0.02 mM, and piperonal dilutions ranged from 0.1 to 0.01 mM in 100 mM sodium phosphate buffer pH 6.0. The respective absorption maxima were determined and plotted against the concentration of cuminaldehyde or piperonal. A linear fit of the data was conducted using the program OriginPro 9.0 (OriginLab Corporation, Northampton, USA) and the molar extinction coefficient was deduced from the fitted data as slope of the regression curve. According to Lambert-Beer-Law with $\mathrm{A} = \varepsilon * \mathrm{c} * \mathrm{d}$, where A is absorbance, ε is the molar extinction coefficient, c is the concentration and d is the path length (1 cm). In a plot with A vs. c, the extinction coefficient is described as the $slope = \varepsilon * d$.

Native PAGE of purified *Pe*AAO2

Blue native PAGE of 5 µg of purified *Pe*AAO2 was carried out using the SERVA*Gel* N Native starter kit (SERVA Electrophoresis GmbH, Heidelberg, Germany) with 4-16 % gel according to the manufacturer's protocol. The gel was stained with Coomassie Brilliant Blue R250.

Influence of pH on enzyme activity

The effect of pH on activity towards *p*-anisyl alcohol, benzyl alcohol, cinnamyl alcohol, cumic alcohol, *trans*,*trans*-2,4-hexadienol, piperonyl alcohol and veratryl alcohol was investigated in 100 mM Britton-Robinson buffer with pH in the range of 2.0 to 10.0. The assay was conducted at room temperature using 1-ml cuvettes with 800 µl of 100 mM Britton-Robinson buffer, 100 µl of 50 mM substrate (10 mM for cumic alcohol) and 100 µl of appropriately diluted *Pe*AAO2. The change of absorbance during product formation was followed using an Ultrospec 7000 photometer (GE Healthcare, Chicago, USA). The corresponding molar extinction coefficients used for calculation were as follows: *p*-anisaldehyde $\varepsilon_{285} = 16{,}980\ \mathrm{M}^{-1}\ \mathrm{cm}^{-1}$ (Guillén et al. 1992), benzaldehyde $\varepsilon_{250} = 13{,}800\ \mathrm{M}^{-1}\ \mathrm{cm}^{-1}$ (Guillén et al. 1992), cinnamaldehyde $\varepsilon_{310} = 15{,}600\ \mathrm{M}^{-1}\ \mathrm{cm}^{-1}$ (Ferreira et al. 2005), cuminaldehyde $\varepsilon_{262} = 2{,}920\ \mathrm{M}^{-1}\ \mathrm{cm}^{-1}$ (this work), piperonal $\varepsilon_{317} = 8{,}680\ \mathrm{M}^{-1}\ \mathrm{cm}^{-1}$ (this work), veratraldehyde $\varepsilon_{310} = 9{,}300\ \mathrm{M}^{-1}\ \mathrm{cm}^{-1}$ (Guillén et al. 1992) and *trans*,*trans*-2,4-hexadienal $\varepsilon_{280} = 30{,}140\ \mathrm{M}^{-1}\ \mathrm{cm}^{-1}$ (Ruiz-Dueñas et al. 2006).

Sequence alignment and homology modelling

The protein sequences of *Pe*AAO (accession number AAC72747) and *Pe*AAO2 (accession number ADD14021) were aligned using the multiple sequence alignment tool Clustal Omega (https://www.ebi.ac.uk/Tools/msa/clustalo/).

Using the homology-modelling server SWISS-MODEL (Waterhouse et al. 2018) and the crystal structure of *Pe*AAO expressed in *E. coli* (Protein data bank (PDB) entry 3FIM, Fernández et al. 2009), a homology model of *Pe*AAO2 was created and the PyMOL Molecular Graphics System (https://pymol.org/2/) was used for visualization.

Supplementary Results

Molar extinction coefficient of cuminaldehyde

The spectra of cumic alcohol and cuminaldehyde were recorded from 200 to 400 nm (Fig. S1).

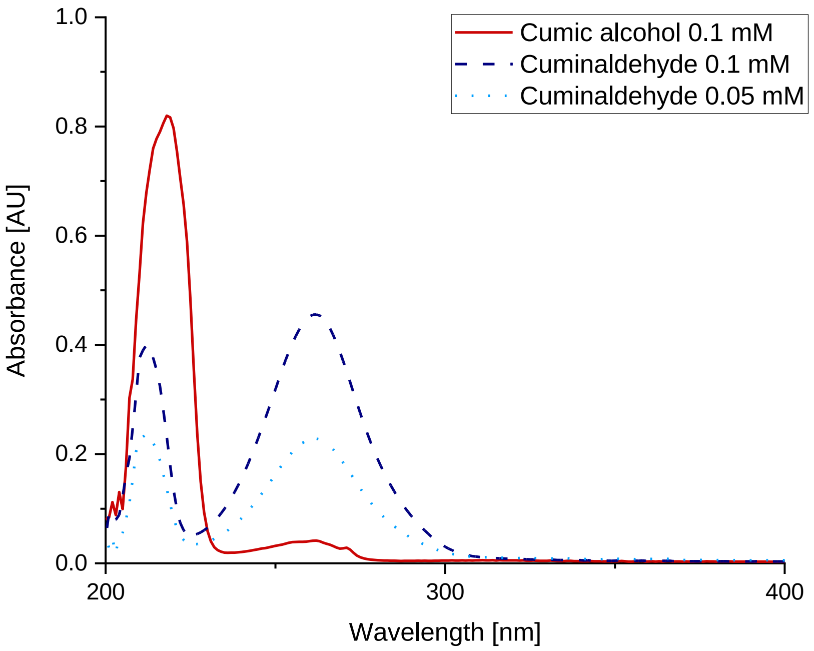

Fig. S1 UV-Vis spectra of cumic alcohol (red, solid line), cuminaldehyde at 0.1 mM (dark blue, dashed line) and cuminaldehyde at 0.05 mM (light blue, dotted line) were recorded in 100 mM sodium phosphate buffer pH 6.0 at 25 °C

The substrate cumic alcohol showed one major absorbance maximum at 218 nm and a minor maximum at 262 nm, whereas the product cuminaldehyde showed a more pronounced absorbance maximum at 262 nm and a second maximum at 212 nm. Therefore, the maximum at 262 nm was used for determination of the molar extinction coefficient of cuminaldehyde.

The absorbance at 262 nm for different cuminaldehyde concentrations was measured (Fig. S2).

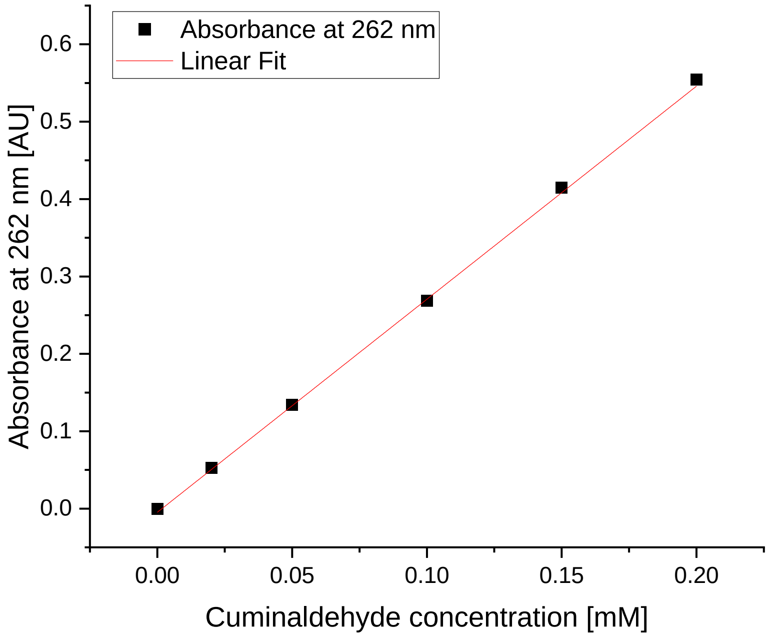

Fig. S2 Correlation of absorbance at 262 nm with cuminaldehyde concentration. The slope of the linear fit represents the molar extinction coefficient ε_{262} in mM^{-1} cm^{-1}. Measurements were done in triplicate from three individual cuminaldehyde stock solutions

The absorbance at 262 nm correlates with the concentration of cuminaldehyde. Using the Lambert-Beer-Law, the molar extinction coefficient ε_{262} of cuminaldehyde under the stated conditions was determined to be $\varepsilon_{262} = 2.92\ mM^{-1}\ cm^{-1}$.

Molar extinction coefficient of piperonal

The spectra of piperonyl alcohol and piperonal were recorded from 250 to 400 nm (Fig. S3).

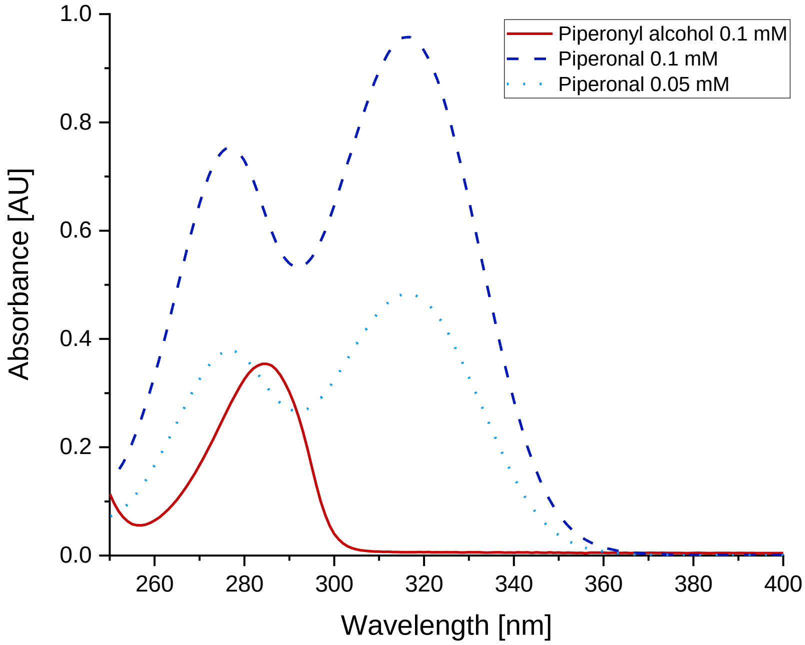

Fig. S3 UV-Vis spectra of piperonyl alcohol (red, solid line), piperonal at 0.1 mM (dark blue, dashed line) and piperonal at 0.05 mM (light blue, dotted line) were recorded in 100 mM sodium phosphate buffer pH 6.0 at 25 °C

The substrate piperonyl alcohol showed a maximum at 285 nm, while the product piperonal showed two strong maxima at 275 and 317 nm. The molar extinction coefficient for piperonal was determined at 317 nm as the absorbance at this wavelength is solely attributed to the aldehyde.

The absorbance at 317 nm for different piperonal concentrations was measured (Fig. S4).

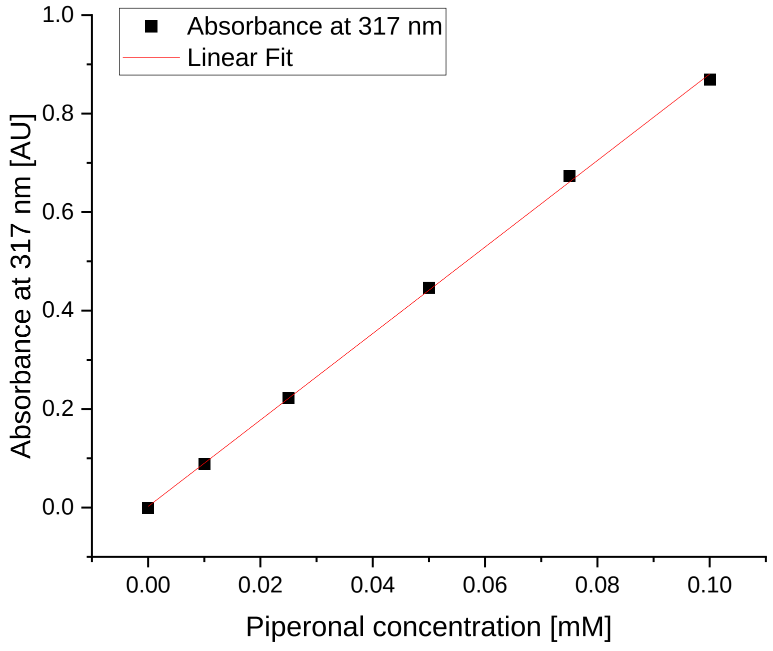

Fig. S4 Correlation of absorbance at 317 nm with piperonal concentration. The slope of the linear fit represents the molar extinction coefficient ε_{317} in mM^{-1} cm^{-1}. Measurements were done in triplicate from three individual piperonal stock solutions

The absorbance at 317 nm correlates with the concentration of piperonal. The molar extinction coefficient for piperonal was determined as described previously for cuminaldehyde. The molar extinction coefficient ε_{317} of piperonal under the stated conditions was determined to be $\varepsilon_{317} = 8.68\ mM^{-1}\ cm^{-1}$.

Native PAGE of purified *Pe*AAO2

The purified *Pe*AAO2 was investigated under non-denaturing conditions in a blue native PAGE (Fig. S5).

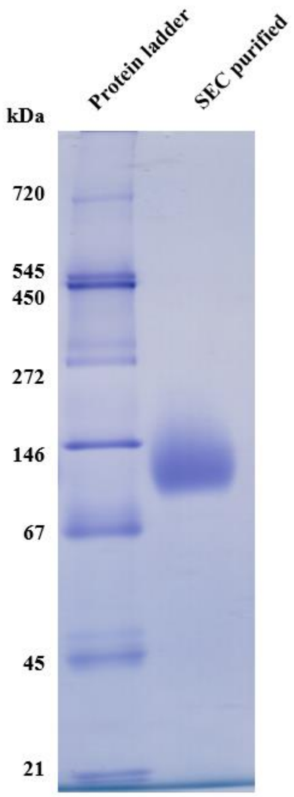

Fig. S5 Gel after Blue Native PAGE of purified *Pe*AAO2. 5 µg of sample were loaded and separated in a 4-16 % gel

Recombinant *Pe*AAO2 moved as a single band between 67 and 146 kDa, and therefore indicates that *Pe*AAO2 is present in a monomeric form.

Influence of pH on activity of *Pe*AAO2

The routinely used buffer in the AAO activity assay mixture was exchanged for 100 mM Britton-Robinson buffer at different pH values. The activity of *Pe*AAO2 towards several substrates at different pH was measured (Fig. S6).

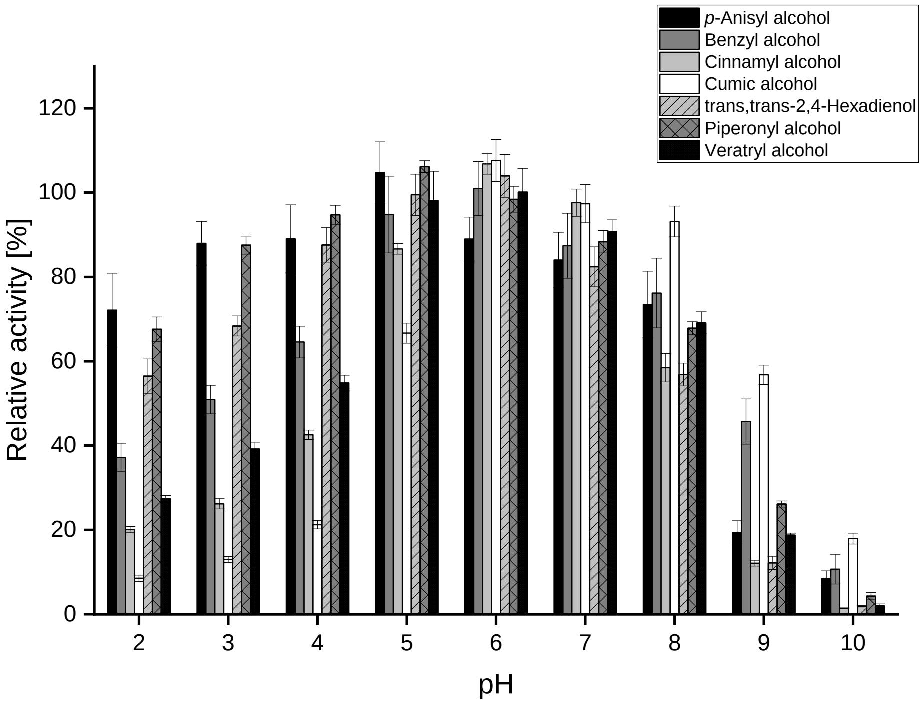

Fig. S6 Influence of pH on activity of *Pe*AAO2. 100 mM Britton-Robinson buffer at the corresponding pH was used instead of the assay buffer - 100 mM sodium phosphate pH 6.0. Values were put in relation to the control experiment with assay buffer. All measurements were done in triplicate

*Pe*AAO2 converted the substrates benzyl alcohol, cinnamyl alcohol, cumic alcohol, *trans,trans*-2,4-hexadienol and veratryl alcohol best at pH 6.0, while for *p*-anisyl alcohol and piperonyl alcohol a slightly more acidic pH 5.0 was best. Relative activities towards *p*-anisyl alcohol and piperonyl alcohol at lower pH were higher compared with other substrates. Overall, the pH activity profile for *p*-anisyl alcohol and piperonyl alcohol appeared shifted towards more acidic pH.

Sequence alignment and homology model

The two *P. eryngii* derived AAOs *Pe*AAO und *Pe*AAO2 differ only in seven amino acid positions (Fig. S7). One of these positions is a potential *N*-glycosylation site (motif Asn-X-Thr/Ser, where X is any amino acid except for proline) in *Pe*AAO2 (residue Asn361), whereas *Pe*AAO lacks this site as an aspartic acid is present instead of asparagine. The catalytically active histidine residues His529 and His573 as well as the main residues involved in regulating substrate accessibility to the active site (Tyr119, Phe424 and Phe528, as described for *Pe*AAO in Fernández et al. 2009) are conserved in both aryl-alcohol oxidases.

```
                       Signal peptide
PeAAO     MSFGALRQLLLIACLALPSLAATNLPTADFDYVVVGAGNAGNVVAARLTEDPDVSVLVLE   60
PeAAO2    MSFGALRQLLLIACLALPSLAATNLPTADFDYVVVGAGNAGNVVAARLTEDPDVSVLVLE   60
          ************************************************************
                                      Asn89                       Tyr119
PeAAO     AGVSDENVLGAEAPLLAPGLVPNSIFDWNYTTTAQAGYNGRSIAYPRGRMLGGSSSVHYM  120
PeAAO2    AGVSDENVLGAEAPLLAPGLVPNSIFDWNYTTTAQAGYNGRSIAYPRGRMLGGSSSVHYM  120
          ************************************************************
                                                 Asn165        Asn178
PeAAO     VMMRGSTEDFDRYAAVTGDEGWNWDNIQQFVRKNEMVVPPADNHNTSGEFIPAVHGTNGS  180
PeAAO2    VMMRGSTEDFDRYAAVTGDEGWNWDNIQQFVGKNEMVVPPADNHNTSGEFIPAVHGTNGS  180
          ******************************* ****************************

PeAAO     VSISLPGFPTPLDDRVLATTQEQSEEFFFNPDMGTGHPLGISWSIASVGNGQRSSSSTAY  240
PeAAO2    VSISLPGFPTPLDDRVLATTQEQSEEFFFNPDMGTGHPLGISWSIASVGNGQRSSSSTAY  240
          ************************************************************
                  Asn249
PeAAO     LRPAQSRPNLSVLINAQVTKLVNSGTTNGLPAFRCVEYAEQEGAPTTTVCAKKEVVLSAG  300
PeAAO2    LRPAQSRPNLSVLINAQVTKLVNSGITNGLPAFRCVEYAEQEGAPTTTVCAKKEVVLSAG  300
          ************************* **********************************
                                             Asn336           Asn352
PeAAO     SVGTPILLQLSGIGDENDLSSVGIDTIVNNPSVGRNLSDHLLLPAAFFVNSNQTFDNIFR  360
PeAAO2    SVGTPILLQLSGIGDENDLSSVGIDTIVNNPSVGRNLSDHLLLPAAFFVNSNQTFDNIFR  360
          ************************************************************
          Asn361                             Asn396
PeAAO     DSSEFNVDLDQWTNTRTGPLTALIANHLAWLRLPSNSSIFQTFPDPAAGPNSAHWETIFS  420
PeAAO2    NSSEFNADLDQWTNTRTGPLTALIANHLAWLRLPSNSSIFQTFPDPAAGPNSAHWETIFS  420
          :*****.*****************************************************
             Phe424
PeAAO     NQWFHPAIPRPDTGSFMSVTNALISPVARGDIKLATSNPFDKPLINPQYLSTEFDIFTMI  480
PeAAO2    NQWFHPAIPRPDTGSFMSVTNALISPVARGDIKLATSNPFDKPLINPQYLSTEFDIFTMI  480
          ************************************************************
                                                   Phe528 His529
PeAAO     QAVKSNLRFLSGQAWADFVIRPFDPRLRDPTDDAAIESYIRDNANTIFHPVGTASMSPRG  540
PeAAO2    QAVKSNLRFLSGQAWADFVIRPFDPRLRDPTNDAAIESYIRDNANTIFHPVGTASMSPRG  540
          *******************************:****************************
                                          His573
PeAAO     ASWGVVDPDLKVKGVDGLRIVDGSILPFAPNAHTQGPIYLVGKQGADLIKADQ  593
PeAAO2    ASWGVVDPDLKVKGVDGLRIVDGSILPFAPNAHTQGPIYLVGERGADLIKADQ  593
          ******************************************::*********
```

Fig. S7 Alignment of *Pe*AAO (accession number AAC72747) and *Pe*AAO2 (accession number ADD14021). The signal peptide comprising the first 27 amino acids is indicated by the blue filled box. Residues in red show differences in both sequences. Residues in purple represent the catalytic active histidine residues His529 and His573. Residues in grey filled boxes are part of the hydrophobic bottleneck, regulating substrate accessibility with Tyr119, Phe424 and Phe528. Residues in green open boxes are possible *N*-glycosylation sites of *Pe*AAO2 with Asn89, Asn165, Asn178, Asn249, Asn336, Asn352, Asn361, Asn396.

The high similarity of both aryl-alcohol oxidases from *P. eryngii*, especially in terms of residues involved in substrate accessibility and catalytically active residues, indicate that both AAOs share similar catalytic properties.

Using the crystal structure of *Pe*AAO expressed in *E. coli* as template (PDB entry 3FIM), a homology model of *Pe*AAO2 was created and the differing residues were marked in purple (Fig. S8). The seven differing residues are located on or near the surface of the protein and the active site is conserved among both AAOs.

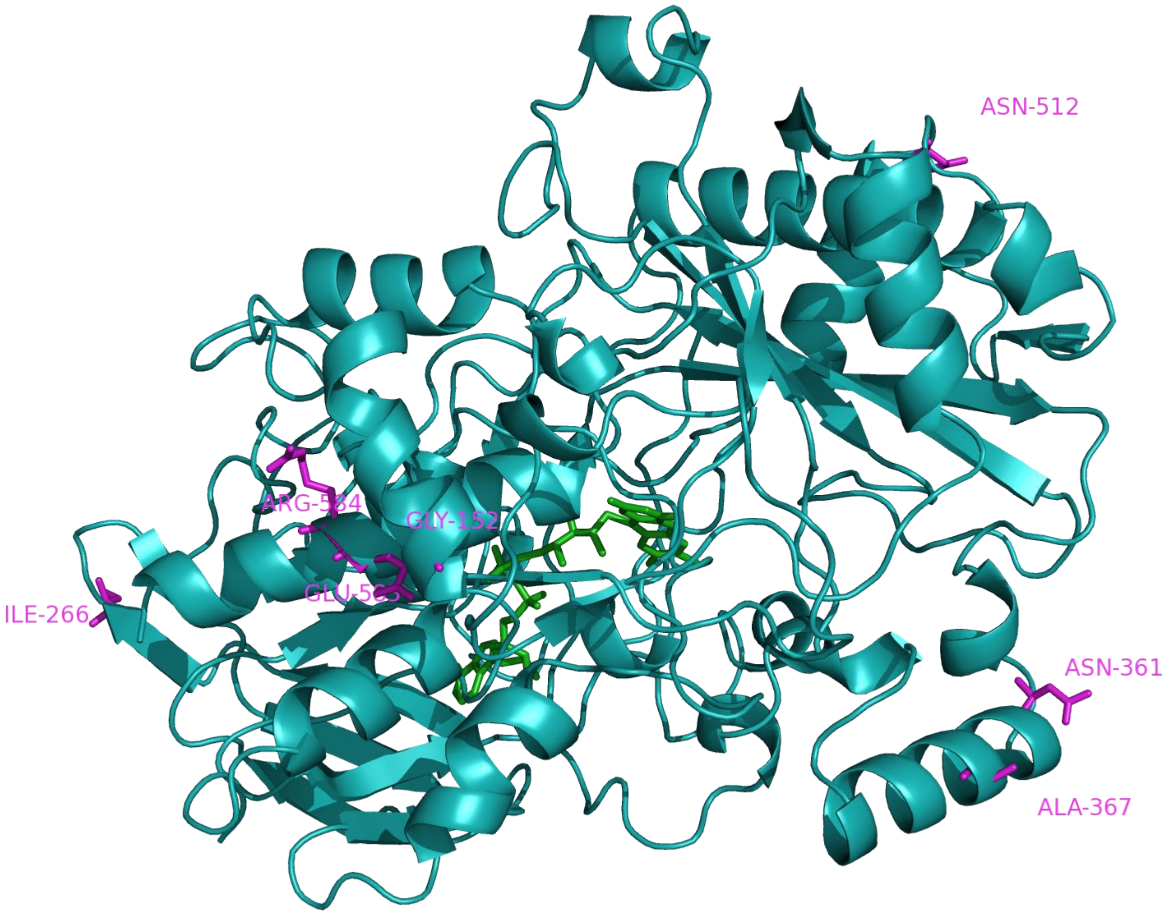

Fig. S8 Homology model of *Pe*AAO2 using the *Pe*AAO crystal structure (PDB entry 3FIM) as template. Residues in purple depict the different positions as compared to *Pe*AAO. Cofactor FAD in green. Numbering including the signal peptide.

References

Fernández IS, Ruíz-Duẽas FJ, Santillana E, Ferreira P, Martínez MJ, Martínez ÁT, Romero A (2009) Novel structural features in the GMC family of oxidoreductases revealed by the crystal structure of fungal aryl-alcohol oxidase. Acta Crystallogr Sect D Biol Crystallogr 65:1196-1205. doi:10.1107/S0907444909035860

Ferreira P, Medina M, Guillén F, Martínez MJ, Van Berkel WJH, Martínez AT (2005) Spectral and catalytic properties of aryl-alcohol oxidase, a fungal flavoenzyme acting on polyunsaturated alcohols. Biochem J 389:731-738. doi:10.1042/BJ20041903

Guillén F, Martínez AT, Martínez MJ (1992) Substrate specificity and properties of the aryl-alcohol oxidase from the ligninolytic fungus *Pleurotus eryngii*. Eur J Biochem 209:603-611. doi:10.1111/j.1432-1033.1992.tb17326.x

Ruiz-Dueñas FJ, Ferreira P, Martínez MJ, Martínez AT (2006) In vitro activation, purification, and characterization of *Escherichia coli* expressed aryl-alcohol oxidase, a unique H_2O_2-producing enzyme. Protein Expr Purif 45(1):191-199. doi:10.1016/j.pep.2005.06.003

Waterhouse A, Bertoni M, Bienert S, Studer G, Tauriello G, Gumienny R, Heer FT, de Beer TAP, Rempfer C, Bordoli L, Lepore R, Schwede T (2018) SWISS-MODEL: homology modelling of protein structures and complexes. Nucleic Acids Res 46(W1):W296-W303. doi:10.1093/nar/gky427
